# Impact of Cancer Stem Cells and Cancer Stem Cell-Driven Drug Resiliency in Lung Tumor: Options in Sight

**DOI:** 10.3390/cancers14020267

**Published:** 2022-01-06

**Authors:** Lourdes Cortes-Dericks, Domenico Galetta

**Affiliations:** 1 Department of Biology, University of Hamburg, 21046 Hamburg, Germany; 2 Division of Thoracic Surgery, European Institute of Oncology, IRCCS, 20141 Milan, Italy; domenico.galetta@ieo.it; 3 Department of Oncology and Hematology-Oncology-DIPO, University of Milan, 20122 Milan, Italy

**Keywords:** cancer stem cells, drug resistance, lung cancer, non-small cell lung cancer, lung cancer therapy

## Abstract

**Simple Summary:**

Lung cancer is still the most common cause of cancer death, worldwide with increasing incidence. In lung cancer management, chemotherapy alone, or combined with radiotherapy induces cell death of the tumor bulk, but not the drug-resistant cancer stem cells (CSCs) causing disease recurrence and, in some cases lead to patient death. This is due to the capacity of CSCs to self-renew and initiate tumor with high heterogeneity that adds complexity to conventional therapy. The reviewed literatures herein affirm the existence of this cell fraction in lung tumors including the CSC- related cellular and molecular mechanisms of drug resiliency. Several novel CSC inhibitors have been tested under in vitro and in vivo conditions, and in few cases in the clinical setting with encouraging results. Nevertheless, in depth investigation is essential to provide more comprehensive data as to the mode of action of these anti-CSC agents particularly, under the clinical setting.

**Abstract:**

Causing a high mortality rate worldwide, lung cancer remains an incurable malignancy resistant to conventional therapy. Despite the discovery of specific molecular targets and new treatment strategies, there remains a pressing need to develop more efficient therapy to further improve the management of this disease. Cancer stem cells (CSCs) are considered the root of sustained tumor growth. This consensus corroborates the CSC model asserting that a distinct subpopulation of malignant cells within a tumor drives and maintains tumor progression with high heterogeneity. Besides being highly tumorigenic, CSCs are highly refractory to standard drugs; therefore, cancer treatment should be focused on eliminating these cells. Herein, we present the current knowledge of the existence of CSCs, CSC-associated mechanisms of chemoresistance, the ability of CSCs to evade immune surveillance, and potential CSC inhibitors in lung cancer, to provide a wider insight to drive a more efficient elimination of this pro-oncogenic and treatment-resistant cell fraction.

## 1. Introduction

Lung cancer is the 2nd most commonly diagnosed malignancy and remains the leading cause of cancer death based on Global Cancer Observatory (GLOBOCAN) 2020 estimates of cancer incidence and mortality [1]. Non-small cell lung cancer (NSCLC) has the highest incidence, about 85%, among all lung cancers, while nearly 15% is represented by small-cell lung cancer (SCLC). NSCLC includes adenocarcinoma, squamous cell carcinoma, and large cell carcinoma [2]. Despite advances in early detection and therapy, the prognosis for lung cancer remains poor due to the late onset of symptoms, and curative resection is often complicated by systemic or local relapse [3]. Nearly 30% of patients with NSCLC have localized disease at the time of diagnosis and undergo curative surgery (early stages, I–II) or, in selected cases, after induction therapies (locally advanced stage, IIIAN2), whereas, in advanced stages (IIIB and IV), resection is generally unfeasible. In the latter, the treatment of choice is the use of platinum-based chemotherapy leading to an initial successful therapy [2], but, in most cases, patients develop secondary tumors that frequently cause lethal relapse [4]. One of the major reasons for therapeutic failure indicates the presence of a niche of cancer cells that cannot be eliminated by standard treatments [5].

In many malignancies, including lung cancer, a small fraction of neoplastic cells is believed to control tumor initiation and progression and can resist conventional therapies. Having similar properties with normal stem cells and their ability to initiate and contribute to tumor development, these cells were termed as CSCs, also known as tumor-initiating cells [6]. CSCs are rare and constitute an insignificant proportion of cells within a tumor mass that are likely to cause cell heterogeneity, sustaining the CSC hypothesis [7]. This model posits that CSCs give rise to highly proliferating progenitor and differentiated cells comprising the bulk of neoplastic tissues that define the histological type of cancer [8]. In solid tumors, including NSCLC, the percentage of tumor-initiating cells comprises a very small subpopulation of <0.02% [9], while <1.0% of the bulk population has been detected in SCLC [10]. However, CSCs do not always constitute a negligible part of a tumor, as a considerable fraction of leukemia-propagating cells has been detected in syngeneic mouse models of lymphomas and leukemias [9].

CSCs exhibit self-renewal, indefinite differentiation properties and can generate heterogeneous lineages of the original tumor when transplanted into a host [11,12]. Being capable of self-renewal, CSCs could go through unlimited cycles of cell division while maintaining their undifferentiated state, a property that is common to embryonic and somatic stem cells [13,14]. However, an aberrant self-renewal leading to uncontrolled amplification directs CSCs to differentiate into a large heterogeneous population of tumor cells with altered phenotypes, resulting in cellular heterogeneity, tumor propagation, and refractoriness to treatment [4,15]. The self-renewal and differentiation properties of CSCs are thought to be strictly regulated by interactions of multiple signaling networks, including the Hedgehog (Hh), Notch and Wnt pathways, and biomolecules, such as cytokines, within the tumor microenvironment [12,16].

CSCs possess intrinsic or acquired resistance to radio- and chemotherapy, resulting in disease recurrence, dissemination, and even death [17,18]. Having the same characteristics as non-malignant stem cells, CSC cycles occur at a much lower rate than those of cancer progenitor cells, which could account for their tolerance to chemotherapeutics directed against dividing cells [19,20]. CSCs identified in lung cancer tissues and cell lines, as well as in tumorspheres, are only partly eliminated when treated with standard chemotherapy and, in some cases, even increase the proportion of CSC surviving cells [21,22,23]. A current study has proposed that CSC-triggered drug tolerance arises between chemotherapy cycles [24] probably due to pre-existing CSC clones that have the capacity to adapt and proliferate thanks to changing factors in the tumor environment and/or in response to chemo-/radiotherapeutics [25]. Notably, chemotherapy could also induce the acquisition of CSC stemness properties in non-stem cancer cells as in gastric and hepatocellular carcinoma cells [26,27], as well as promoting the transformation of non-cancer stem cells to CSCs in breast cancer cells after radiotherapy [28]. The intrinsic resiliency of CSCs in lung cancer is diverse and may be attributed to the following factors: quiescent state with low proliferation rate, [29], activation of drug-efflux processes by the ATP binding cassette (ABC) transporters [30,31], overexpression of DNA-repair mechanisms [18,32], decreased programmed cell death [33], acquisition of an epithelial-to-mesenchymal (EMT) phenotype [34] and ALDH activity [35].

CSCs are endowed with the ability to escape innate and adaptive immune control, a feature known as an immune privilege [36]; thus, they can shape the tumor microenvironment (TME) into an immunosuppressive, pro-tumorigenic niche [37]. Within the TME, specialized areas such as CSC niches consisting of non-cancerous stromal cells and cancerous non-stem cells preserve CSC status and plasticity and safeguard these cells from immune attacks [19]. This dynamic interaction of CSCs with microenvironmental components enables them to overcome infiltrating immune cells, thus assuring their cell viability and development into a cognizable disease [38,39].

The mechanisms of drug resistance supposedly exerted by CSCs in lung cancer are yet partially resolved owing to their multiple lines of defense. In this review, we provide evidence of the presence of CSCs, CSC-associated mechanisms of drug/treatment resiliency, the capacity of CSCs to escape immune control, and diverse CSC-inhibitory agents to provide a broader knowledge in the elimination of this pro-tumorigenic and therapy-resistant cell subpopulation.

## 2. Identification of CSCs in Lung Cancer

The existence of CSCs in lung cancer has been detected back in 1981 by Carney and colleagues [40]. This group found that about 1.5% of tumor cells from lung adenocarcinoma patients were able to form colonies when cultured in vitro—hence, termed as colony-forming cells—and could also reconstitute tumors when inoculated into nude mice. Since then, studies followed affirming the existence of putative or bona fide CSCs in lung cancer using different CSC surface markers, alone or in combination with other stemness-associated indicators of pluripotency genes.

CD133 (prominin-1), a 5-transmembrane glycoprotein, is an established marker for CSC fraction in lung cancer. For instance, the flow cytometry cell sorting (FACS)-enriched CD133^+^ cells from human NSCLC tissues and lung cancer cell lines exhibited self-renewal, drug- and radiotherapy resistance and co-expressed ABCG2, a multidrug resistance marker and octamer-binding transcription factor 4 (Oct4), a marker for stem cell pluripotency [41]. High levels of CD133^+^ co-expressing epithelial antigen (ESA) were detected in NSCLC tissues, manifesting high tumorigenicity in a SCID mouse model, high expression of genes associated with stemness, and the ability to efflux drugs [22]. In the SCLC and NSCLC specimens, a subpopulation of tumorigenic cells expressing CD133 were identified exhibiting tumor-forming abilities and could generate lung tumor xenografts identical to the original tumor [42]. Likewise, tumorspheres grown from A549 cancer cells expressed high levels of CD133, self-renewal, increased drug insensitivity, and high tumorigenicity in vivo and expressed CSC-related genes, Oct 4, and sex-determining region Y-box (Sox2), highly indicating the presence of CSCs [43]. One study demonstrated that CD133^+^ cells from NSCLC patient specimens acquired a higher expression of CD133 when grown as tumorspheres and were more tumorigenic in SCID mice. Intriguingly, Cui et al. [44] found that CD133 only served as a CSC marker in SCLC cells H446, but not in the five tested NSCLC cell lines. A separate work observed that CD133^+^-sorted cells from SCLC patients elicited chemoresistance and high tumorigenicity and had high levels of AKT/PKB and BCl-2. Here, CD133 expression increased after chemotherapy in mouse and human SCLC, with the latter having been confirmed in clinical specimens from a patient receiving chemotherapy—providing evidence of the intrinsic tolerance of CSCs to chemotherapeutics [23].

ALDH is one of the established CSC-associated markers in lung cancer. Aldehyde dehydrogenases (ALDHs) are a group of nicotinamide-adenine dinucleotide phosphate-positive [NAD(P)^+^]-dependent enzymes that catalyze the oxidation of aldehydes to carboxylic acid [45]. In the SPC-A1, lung cancer-derived tumorspheres, high ALDH1 positivity, high cloning efficiency, and profound tumorigenicity were observed, indicating that ALDH1 can serve as a specific marker for CSCs in human lungs [46]. The enriched ALDH cell fraction from human lung cancer cells exhibited self-renewal properties and drug insensitivity; co-expressed CD133; and could recapitulate the heterogeneity of the parental cancer cells representing the basic features of CSCs [47]. Another study found that FACS-sorted ALDH bright (ALDH^br^) and dim (ALDH^lo^) cells were able to generate primary tumors in mice, but the secondary and tertiary tumors from ALDH^br^ developed faster and bigger than those from ALDH^lo^, indicating the fundamental feature of CSCs [48]. Li et al. [49] found higher ALDH1A1 expression in lung adenoma cells isolated from A549 (A549s) than that of parental A549 cells. Additionally, they showed that ALDH1A1 maintained stemness of A549s by improving clonogenicity and inhibition of the cell cycle. A panel of lung cancer cell lines and NSCLC patient tumors was found to contain a cell fraction with augmented levels of ALDH activity associated with ALDH1. Here, isolated ALDH^+^ cells manifested high tumorigenicity, clonogenicity, and self-renewal capacity, as opposed to ALDH^−^ cells, demonstrating that ALDH could select for NSCLC stem-like cells [50].

CD44, a cell-surface adhesion receptor has been used, in combination with ALDH, to identify potential CSCs in lung cancer. In the different lung cancer cell lines and patient-derived lung cancer cells, an ALDH^hi^/CD44^hi^ subset manifested the highest attributes of stem cell phenotypes, higher invasion capacities, expression of pluripotency genes, epithelial-mesenchymal transition (EMT) transition genes, and stronger in vivo tumorigenicity than other types of sorted ALDH/CD44 and unsorted cells, indicating the competence of ALDH/CD44 in identifying and maintaining tumor-initiating cells [51]. Nishimo et al. [52] reported that, in several lung adenocarcinoma cells lines, CD44v^high^/ALDH^high^ and CD44v^high^ ALDH^low^ cells were enriched with CSC phenotypes, the latter being more proliferative and more tolerant to drug treatments, whereas CD44v^high^/ALDH^high^ elicited higher tumor-sphere formation and stronger generation of primary and secondary tumor xenografts. Notably, lung cancer cells from clinical samples contained a significantly higher percentage of ALDH1A1^+^ cells that were resistant to epidermal growth factor receptor (EFGR) trypsin kinase inhibitor (TKI) and chemotherapy, manifesting one of the fundamental properties of CSCs [53]. A novel triple marker, EpCAM/CD166/CD44 was identified in A549 cancer cells, bearing high proliferation activity, clonogenicity, self-renewal property, and chemoresistance to cisplatin and 5-fluorouracil, owing to high ALDH activity in this subpopulation [54]. Further, urokinase plasminogen activator receptor (uPAR)^+^ cells in six human SCLC cell lines co-expressing CD44 and MDR1 were found resistant to conventional chemotherapies [55].

Side population (SP) cells, initially described in hematopoietic stem cells, have the ability to efflux the DNA-binding dye Hoechst 33342 outside of the cell membrane via the ATP-binding cassette (ABC) transporter [56]. In lung cancer, SP cells have been used to enrich and characterize a cell fraction exhibiting the fundamental characteristics of CSCs, particularly, their tolerance to chemotherapeutics. The enriched SP cells in several lung cancer cell lines (H460, H23, HTB-58, A549, H441, and H2170) showed high tumor-initiating properties, as well as elevated expression of the ABCG2 and ABC transporters that were insensitive to several chemotherapeutics [57]. The FACS-sorted SP cells from the A549 lung cancer cell line demonstrated increased colony-forming ability [58] and cell invasiveness, including high tumorigenic potential [59]. In another study, A549-enriched SP cells, when treated with doxorubicin (DOX) and methotrexate (MTX) manifested active regeneration and high anti-apoptotic abilities [60]. The SP cell fraction in three SCLC cell lines was found to exhibit high proliferation and self-renewal properties, CSC- and drug resistance-associated genes, and increased in vivo tumor reconstitution in a mouse model [10]. A mechanistic study showed that the constitutive expression of redox-sensing NRF2, in addition to ABCG2, is involved in eliciting chemoresistance to chemotherapeutics in lung cancer SP cells [61].

CD90, a cell adhesion molecule, known as thymocyte differentiation antigen-1 (Thy-1), has been reported as a CSC-associated marker for lung cancer. Yan and coworkers [62] isolated CD90^+^ cells from A549 and H446 lung cancer cell lines, which exhibited higher tumorigenicity, proving its competence as a potential CSC biomarker in lung cancer.

CD166, also named activated leukocyte cell adhesion molecule (ALCAM) [63], is supposedly an “inert” CSC marker for NSCLC, according to Zhang and colleagues [64]. These authors found that FACS-enriched CD166^+^ cells manifested self-renewal properties and the ability to form xenograft tumors, which were not observed in the CD166-negative cell fraction. However, both enriched CD166 and unsorted cells formed tumors, the former requiring only 5000 cells, whereas the latter needed 500,000 cells. The silencing of CD166 in patient-derived tumor cells generated no significant effects and showed no association between CD166 expression and patient survival, hence the proposal that CD166 is an inert CSC marker of NSCLC. Tachezy and coworkers [65] also showed uncertainty in considering CD166 as a CSC marker in NSCLC. Here, they found that CD166 levels in primary NSCLC specimens are associated with smaller tumors without lymph node metastasis and had no prognostic effect on patient survival, thus indicating CD166 as an atypical CSC marker.

## 3. CSC-Associated Cellular and Molecular Mechanisms of Chemoresistance

**Figure 1:**
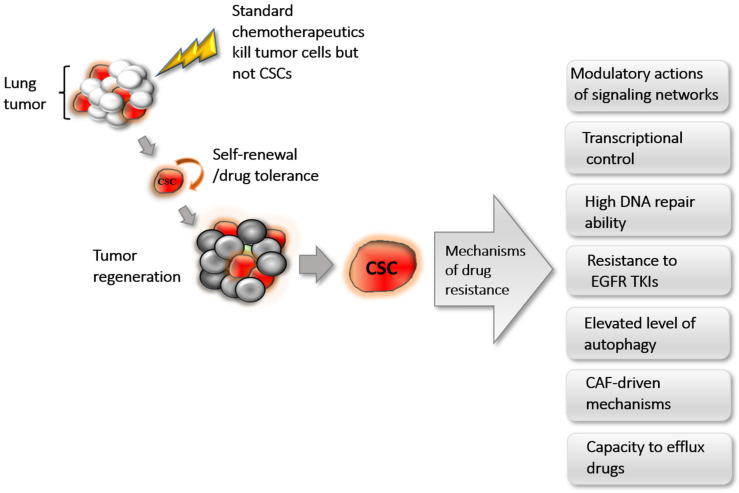
The CSC-mediated mechanisms of drug resistance in lung cancer are multifactorial. CSCs are known to escape conventional therapy and are able to regenerate the bulk of the tumor, causing disease recurrence. The literature reviewed herein asserts that CSCs are recalcitrant to effective treatment because of the modulatory actions of diverse signaling networks, aberrant transcriptional control, high DNA repair ability, resistance to EGFR TKIs, elevated autophagy, presence of cancer-associated fibroblast (CAF)-driven mechanisms, and ability to efflux drugs through ABC transporters in SP cells. CSC, cancer stem cells; EGFR, epidermal growth factor receptor; TKIs, tyrosine kinase inhibitors; SP, side population; ABC, ATP-binding cassette.

Lung cancer research has provided evidence of diverse cellular and molecular mechanisms (Figure 1) regulating the activities of CSCs that assert their critical implication in resiliency to conventional chemotherapeutics.

### 3.1. Modulation through Signaling Networks

The Notch pathway is one of the potential therapeutic targets of CSCs in solid tumors. A current study [66] found that chemoresistant NSCLC patients had upregulated Notch3 expression, which was related to poor prognosis and had augmented levels of CSC markers, ALDH1A1 and CD44, correlating with Notch3 expression in lung cancer biopsies. Here, autophagy was also activated in drug-resistant lung cancer cells, thus postulating a critical role of Notch3 not only in enhancing the stem-like properties but also in the activation of autophagy. A subsequent report discovered a subset of lung CSCs (LCSCs) from NSCLC samples exhibiting a CD166^+^CD49f^hi^CD104^−^Lin^−^ phenotype, tumorigenicity in vivo, self-renewal, and sphere-forming properties. In this work, Notch1 was found essential for the maintenance of self-renewal and preservation of LCSCs from cisplatin-induced cell death via the transcription factor HES1 and through a specific HES1-independent pathway [67].

In enriched NSCLC 95-D cell-derived CD133^+^ lung CSCs (95-D LCSCs), the overexpression of neural EGFR like 1 (NELL1) conferred a decrease in colony formation and cell invasion, as well as an increase in chemosensitivity to carboplatin and cisplatin. At the molecular level, NELL1 diminished the levels of phospho-Met (p-MET), Notch 3, and HES1, suggesting a NELL1-mediated inhibition of the c-MET-Notch signaling that stimulates 95-D LCSCs cell differentiation, resulting to lower invasion, migration, and proliferation properties [68].

The drug response of CSC epithelial-type (E-cadherin^high^/CD133^high^) cells with higher sphere-forming capacity and chemoresistance has been recently elucidated. This investigation found that epithelial-type CSCs had upregulated the MyoD family inhibitor domain (MDFIC), while a knockdown of this domain sensitized epithelial-type CSCs to chemotherapeutic drugs. Mechanistic investigations disclosed that an MDFIC p32 isoform interacted with the Axin/GSK-3/ß-catenin complex, thus stabilizing ß-catenin. Meanwhile, the silencing of ß-catenin resulted in a decrease in MDFIC-mediated chemoresistance, indicating that chemoresistance in the epithelial-type CSCs is due to an upregulation of MDFIC that eventually increases ß-catenin activity [69]. In a previous study, the functional role of Wnt/ß-catenin in maintaining highly resistant lung CSCs appeared to be justified by silencing ß-catenin, which promoted sensitivity to chemotherapy, and by using Wnt antagonist, PP and EGFR-TKIs, which decreased metastasis and induced apoptosis [70].

The NSCLC-derived CD166^+^ cell fraction (CD166^+^ LCSCs) expressed low levels of Solute Carrier Family 27 Member (SLC27A2), correlating to poor patient survival and poor chemo-response, as well as expressing high levels of Bmi1 and ABCG2. Mechanistic analyses showed that reduced SLC27A2 conveyed chemoresistance by negatively modulating Bmi1-ABCG2 signaling, hence asserting ABCG2 as a direct target of Bmi1 [71].

Cisplatin (CCDP)-resistant A549 lung cancer cells (A549/CCDP) acquired the EMT phenotype linked with migratory and invasive abilities, as well as pronounced CSC properties. The molecular mechanisms for these observations were found to be regulated by the AKT/β-catenin/Snail signaling network [72]. A separate work showed that decreased apoptotic response of lung CSCs to superoxide, cisplatin and gemcitabine was mediated by inactivation of caspase-9 and caspase-2, as well as increased activation of p38MAPK, MAPKAPK2, and Hsp27 [73]. Another chemoresistance mechanism of NSCLC to cisplatin is through Doublecortin-like kinase 1 (DCLK1) via an ABC subfamily member 4 (ABCD4)-dependent mechanism [74].

The activation of D2 dopamine (DA) receptors in NSCLC CD133^+^ CSCs could significantly restrain their proliferation, clonogenic ability, and invasiveness by repressing the extracellular signal-regulated kinases 1/2 (ERK1/2) and AKT, including the downregulation of Oct4 and metalloproteinase-9 (MMP-9) secretion by these cells [75].

### 3.2. Transcriptional Control

The embryonic transcription factor SOX9 is considered a crucial regulator of acquired drug resistance in NSCLC. Voronkova and colleagues [76] found an increased SOX9 expression after cisplatin treatment of NSCLC cells, whereas silencing of SOX9 increased their sensitivity to this drug. SOX9 was also found to promote the stem cell-like phenotype and increase the ALDH activity in NSCLC cells; further, it was proved that ALDH1A1 is a direct transcriptional target of SOX9. In this context, the authors proposed the SOX9–ALDH axis as a CSC regulator that may serve as a prognostic marker of chemoresistance and drug target in NSCLC.

The regulatory actions of Forkhead box C1 (FOXC1) on CSC-like characteristics in NSCLC have been recently investigated. This study found that the silencing of FOXC1 reduced CD133^+^ cells, self-renewal ability, and expression of CSC-related genes (OCT4, NANOG, SOX2, and ABCG2); increased cisplatin and docetaxel sensitivity; and reduced gefitinib resistance. Further findings showed that β-catenin is a direct transcriptional target of FOXC1 and that overexpression of β-catenin reversed the inhibition of the CSC properties induced by FOXC1 knockdown. Meanwhile, the silencing of β-catenin decreased the CSC phenotype mediated by FOXC1 overexpression, suggesting that FOXC1 induces CSC-like characteristics in NSCLC by promoting β-catenin expression [77].

The *GSTP1* gene codes for glutathione S-transferase pi 1 (GSTP1), a phase II detoxification enzyme that is directly implicated in the detoxification of cisplatin by generating cisplatin–glutathione adducts [78]. An investigation using parental Lewis lung carcinoma lung (LLC-parental) and human lung cancer cells (H1299)-derived CSCs revealed that the cisplatin resistance of these CSC compartments is through the transcriptional activation of *GSTP1* by MEK/ERK signaling [79].

### 3.3. Cancer-Associated Fibroblast (CAF)-Driven Mechanisms

CAFs are one of the cancer stromal cell types in the tumor environment that have emerged as major players in mediating resistance to chemotherapeutics via diverse mechanisms [80,81]. CAFs produce biomolecules, which are in a tight crosstalk with CSCs regulating their self-renewal, plasticity, and refractoriness to chemotherapeutics [82,83]. In NSCLC, CAFs maintained CSC stemness in a paracrine manner, as observed in a co-culture of CAFs with CSCs. Herewith, CAFs generated insulin-like growth factor-11 (IGF-11) that activated insulin growth factor 1 receptor (IGFR) on CSCs, which, in turn, modulated the IGF-II/IGF1R/Nanog signaling pathway to maintain CSCs’ stemness [83]. A cell fraction of CAFs exhibiting CD10 and GPR77 phenotypes correlated with poor survival, as well as chemoresistance, in breast and lung cancer. In particular, CD10^+^GPR77^+^ CAFs promoted tumor formation and drug resiliency by providing a survival niche for CSCs. Mechanistically, these observations revealed that constant activation of NF-κB coupled with p65 nuclear retention in the CD10^+^GPR77^+^ CAFs is necessary to maintain a paracrine secretion of IL-6/IL-8 and provide a CSC niche for CSCs [84]. An additional mechanism of CAFs to provide a protective niche for CSCs under chemotherapeutic treatment is through increased levels of ABCG2 in cancer cells—A known mechanism of CSC drug resistance [85].

### 3.4. High DNA Repair Ability

The DNA damage response of NSCLC-derived lung CSCs after exposure to chemotherapeutic drugs has been accounted for as a cell cycle arrest, thus allowing DNA damage repair and consequent cell survival. This work discovered that the earliest molecular event in this process was the activation of the DNA damage checkpoint protein kinase 1 (Chk) independent of their p53 status [86]. A separate study discovered that lung cancer stem cells (LCSCs) and differentiated LCSCs (dLCSCs) responded differently to cisplatin chemotherapy. LCSCs were found more resistant to cisplatin-induced cytotoxicity than dLCSCs because of their ability to reduce the intracellular accumulation of cisplatin and higher ability to repair cisplatin–DNA interstrand crosslinks [87].

### 3.5. Resistance to EGFR TKIs

Resistance to EGFR tyrosine kinase inhibitors (TKIs) is one of the major obstacles in lung cancer therapy. An association between ALDH1A1 and EGFR TKI resistance in lung CSCs has been investigated, wherein ALDH1A1^+^ lung cancer cells were found more resistant to gefitinib than the corresponding ALDH1A1^−^ fraction [53]. Further findings also observed that PC9/gef cells gefitinib-resistant lung cancer cells) exhibited higher percentages of ALDH1A1 than PC9 cells (gefitinib-sensitive cells). In corroboration, clinical specimens from patients that were resistant to EGFR TKI also manifested significantly high levels of ALDH1A1^+^ cells [53]. *Oct4*, a CSC-associated gene, has also been reported to play a crucial role in the maintenance of resistant NSCLC CSCs with EGFR mutation to gefitinib. Transfection of Oct4 into PC9 and HCC827 cells bearing EGFR mutation significantly increased CD133^+^ gefitinib-resistant cells and sphere-formation under high concentrations of gefitinib in vitro. More importantly, tumor specimens from EGFR mutant NSCLC patients with acquired resistance to gefitinib also showed high Oct4 levels [88].

### 3.6. Elevated Autophagy

Autophagy is a cellular survival mechanism, which is induced by cancer therapy; thus, it plays a critical role in the regulation of drug resistance, particularly in CSCs [89,90]. Indeed, Yang and coworkers [58] measured increased levels of autophagy in A549-enriched SP cells during cisplatin treatment causing drug resistance, whereas the inhibition of autophagy promoted cisplatin-induced apoptotic effects, suggesting its implication in potentiating the cytotoxic activity of cisplatin in A549 cells. In parallel, cisplatin treatment of NSCLC specimens and A549 cell line also showed an increased number of CD133^+^ cells and exhibited higher autophagy levels, which was reversed by chloroquine (QC). Thereafter, the combination of cisplatin and QC proved to be a better treatment than individual agents in promoting the efficacy of cisplatin [91].

### 3.7. Drug Efflux through ABC Transporters

The ABC transporter proteins ABCB1 (MDR1/P-glycoprotein), ABCC1, and ABCG2 permit the transmembrane transport of different substrates, including chemotherapeutic agents; thus, they are implicated in multidrug resistance (MDR) in different types of tumors, such as lung cancer—reviewed in [92]. SP cells do not merely represent a cell fraction enriched with CSC phenotypes but are also implicated in MDR, mainly attributed to high levels of ABCG2, provoking an active efflux of drugs from within the cells [93]. On this basis, the constitutive expression of redox-sensing NRF2, in addition to ABCG2, was found to be involved in eliciting tolerance to chemotherapeutics in lung cancer SP cells [61]. When A549-enriched SP cells were treated with doxorubicin (DOX) and methotrexate (MTX), these cells exhibited active regeneration and high anti-apoptotic abilities, as compared to non-SP cells. The resiliency of SP cells to DOX and MTX was accounted for having augmented levels of ABCG2 and ABCC2 [60]. Accordingly, high expression of four major ABC transporters (ABCA2, MDR1, MRP1, and ABCG2) was detected in the SP fraction of six human lung cancer lines, which exhibited high drug resistance to multiple chemotherapeutics, several of which are conventionally used as first-line therapy for lung cancer [57]. Another study disclosed that the drug resiliency of NSCLC to cisplatin is through Doublecortin-like kinase (DLCK1) via an ABC subfamily member 4 (ABCD4)-dependent mechanism [74].

## 4. CSC Inhibitors in Lung Cancer

Treatment failure of conventional lung cancer therapy can likely be augmented by precise elimination of the CSC cell fraction. On this account, small molecule inhibitors, as well as natural compound-based inhibitors, acting on specific CSC subpopulations or downstream molecules of CSC-associated pathways have provided encouraging results, as presented in Table 1 and Table 2, respectively.

## 5. Immunological Attributes of CSCs

The “cancer immunoediting hypothesis” advocates that both tumor cells and CSCs possess various strategies to escape immune attacks, resulting in reduced immune recognition, activation of oncogenic pathways that lead to intensified tolerance to cytotoxic effects of immunity, absence of tumor antigen expression, and modulation of a protective immunosuppressive microenvironment [128]. As the CSC model is increasingly accepted in the field of cancer immunity, some argue that CSCs may be the key players in all three phases of the immunoediting process [36,129], which is characterized by three Es (elimination, equilibrium, and escape phases).

During the elimination phase (early stage of tumor development), CSCs do not receive the influence of the immune system, remaining protected from it. In particular, cooperating with adaptive immunity (T and B lymphocytes), the innate immune cells, including neutrophils, macrophages, natural killer (NK), and dendritic cells, attack the tumor mass but fail to eradicate CSCs thanks to their ability to (a) enter quiescence, (b) activate anti-apoptotic pathways and (c) upregulate the “don’t eat me” signal CD47 [130]. These properties may help CSCs remain detached in their niches safe from the highly active and functional immune system during this phase. In the next stage, the equilibrium phase, CSCs may escape immune destruction by modifying their features, enfolding their low multiplication rate and high resistance to cell death/killing, and increasing molecular alterations, which helps them to enter the next phase. During the last stage, the escape phase, CSCs secrete cytokines, chemokines, and soluble factors that activate different pathways, aiming at tipping the balance towards immune tolerance, to suppress and edit the immune system and create a pro-tumoral niche [19,131]. In this phase, there is a decreased expression of human leucocyte antigen I (HLA-I) molecule when cells are enriched with stem-related markers isolated from patients with locally advanced head and neck squamous cell carcinoma receiving chemotherapy treatment [132]. It has been observed that CSCs isolated from the lung cancer cell line exhibited a lower expression of MHC-I than their differentiated counterparts [14]. The reduced expression of HLA-I confers CSC-like properties and protection from T-cell recognition [133]. In particular, this suppressed expression, if associated with detectable natural killer group 2D (NKG2D) ligand, can lead to an increased susceptibility of CSCs to natural killer (NK) cells [134]. Other mechanisms of immune escape were identified and include (a) the heterogeneous expression of the immune checkpoints (e.g., Cytotoxic T-Lymphocyte Antigen 4—CTLA4), B7H3 and V-set domain-containing T-cell activation inhibitor 1 (B7-H4) and Programmed death-ligand 1 (PD-L1) [135]; (b) the down-modulation of innate immune pathways (e.g., the toll-like receptor 4—TLR4) [136,137]; and (c) the components of the signal transducer and activator of transcriptor 3 (STAT3) pathway [135].

An important feature of CSCs is their ability to switch between dormant and proliferating states [138]. CSCs have the capacity to exit the cell cycle and remain in the G0 phase in a quiescence status. CSCs can escape host anti-tumor immunity by three different modalities: (1) preventing immune detection, (2) preventing immune activation, and (3) activating immune suppression [139]. The ability of dormant CSCs to evade T and NK cells is due to the downregulation of the MHC-I complex [140] and UL16 binding protein (ULBP) ligands [141], respectively. The immunosuppressive functions exerted by CSCs, including the expression of immune checkpoints such as PD-L1 and B7.1, allow them to prevent T cell activity and cancer dormancy. In addition, other mechanisms, such as the genetic inactivation of the oncosuppressor caspase 8 (CASO8) and death receptor Fas cell surface death receptor (FAS) [142], allow dormant CSCs to escape NK- and T-cell-mediated apoptosis.

Among the immune cell population involved in the control of CSCs, some subtypes of T cells deserve attention, namely, (a) regulatory T cells (Tregs), (b) T helper 17 cells (Th17), and (c) CD8+ T cells. Tregs are a subset of CD4+ immune T cells that, in physiological conditions, guarantee tolerance to self-antigens and prevent/suppress autoimmune reactions, while, in cancer, they supply tumor progression and tumorigenesis by impairing host immune defenses. Th17 are a subset of CD4+ T cells characterized by the production of IL-17 and are also the key mediator of cancer development by their characteristic of both tumor-promoting and tumor-suppressing activities [143].

CD8+ T cells are a subtype of T cells and the main effectors of cell-mediated adaptive immune responses. Activated CD8+ T cells produce an effector cytokine, the interferon-gamma (IFN-γ) that induces NSCLC stem cells in a dose-dependent manner [143]. CSCs dialog with CD4+, CD8+ T cells, and Tregs and actively participate in the induction of an immunosuppressive milieu, which fuels their immune evasion and malignant potential [143].

## 6. Immunotherapy against CSCs

CSCs can shape the tumor microenvironment by attracting immunosuppressive innate cell subsets and inhibiting effector T cells. On the other hand, stromal cells and infiltrating immune cells support CSCs’ tumorigenicity, self-renewal, and metastasis. Moreover, the capability of CSCs to resist conventional cancer therapies is most likely due to their high capability to repair DNA damage and proliferate slowly. The combination of radiation commonly used to treat many types of cancer, and immunotherapy is considered a promising therapeutic tool [144]. This combination is expected to have synergistic effects, stemming from both local and systemic tumor control, due to the interactions between radiation and the immune system [145].

Combination immunotherapies would be an ideal approach to restore anti-tumor immunity against CSCs. Active STAT3 signaling plays an important role in CSCs/immune cells interaction, including the effect of IL-6 and IL-17 on the stemness and suppressive (e.g., PD-L1 expression) properties of CSCs [146]. Many of these effects could be reversed by the inhibition of STAT3. This renders this molecule an attractive therapeutic target to block CSC-associated tumor immune evasion [102]. For example, the inhibitor OPB-51602 is showing promising effects against non-small cell lung carcinoma [147].

Another possible target for therapy is the SIRPα ligand CD47 that is overexpressed by CSCs [148]. Indeed, several studies have shown that blocking CD47 results in increased phagocytosis of CSCs by macrophages as tested in ongoing clinical trials [149]. Moreover, CSCs might be eliminated by using specific immunotherapeutic approaches, such as chimeric antigen receptor- or T cell receptor-engineered T cells (CAR T-cells), drug-conjugated monoclonal antibodies, DC-based therapeutic vaccines, and targeting antigens that are characteristically expressed by CSCs [150,151,152].

Taken together, these results confirm that the relationship between the immune system and a tumor is intricate. The immune system can play pro-tumorigenic and anti-tumorigenic roles. Innate and adaptive immune cells can kill tumor cells, but they often fail in destroying CSCs, because CSCs can shift their phenotypes, as well as modulate the function of immune cells. CSCs can produce, express or secrete factors and surface proteins that suppress the immune cells’ ability to eradicate CSCs. In addition, CSCs recruit immune cells to the tumor microenvironment that have immunosuppressive activity, thus promoting their survival. Hence, immune cells contribute to the maintenance, growth, development, and migration of CSCs and, ultimately, the development of the tumor. Therefore, targeting CSCs by immunotherapy-based approaches, such as equipping cells with CAR, would aid in eliminating these tumor-initiating cells.

## 7. Conclusions and Future Insights

Based on this review, CSCs are cancer cells believed to be the root of sustained tumor growth that underlies chemoresistance and tumor relapse. The presence of CSCs has been largely demonstrated in lung cancer using surface markers, alone or in combination with other stemness-associated indicators or CSC-associated genes. Evidence of diverse CSC-triggered cellular and molecular mechanisms, such as high DNA repair ability, elevated autophagy, capacity to efflux drugs, resistance to EGFR TKIs, and CAF-promoting mechanisms, among others, elucidates their crucial implication in drug tolerance to conventional chemotherapeutics. Several CSC inhibitors (in some cases, combined with standard chemotherapy) targeting associated molecules of these signaling events or biological processes have proven successful in the suppression of CSC activities. In addition to their innate refractoriness to chemotherapeutics, CSCs can escape cancer immune surveillance by their capacity to evade immunoediting and could escape host anti-tumor immunity by preventing immune detection and activation and, finally, the activation of immune suppression.

Most of the data presented herein were obtained under in vitro conditions using lung tumor cell lines. Tumor-derived cell lines are frequently used in cancer research because they represent a fraction of pure tumor cells without contaminating stromal or inflammatory cells; however, they may also represent minor tumor cell subpopulations not characteristic of the original tumor. Of note, the absence of vascularization, stromal, immune, and inflammatory cells in tumor cell lines limits the in vivo study of a complex interaction of specific compound(s) with other molecules and cell types. Although in vitro testing using lung cancer cell lines has been a fundamental part of preclinical research to understand the cellular and molecular basis of drug resistance and other characteristics of tumor cells, the interpretation of data from these projects should be performed with great caution.

Although we have come a long way in understanding the signals that drive cancer growth and how those signals can be targeted, the effective control of lung cancer remains a key scientific and medical challenge, particularly because of CSCs. Using conventional treatments to target CSCs is unlikely because of their inherent phenotypic and functional heterogeneity and aberrant signaling networks that modulate CSC stemness. Nevertheless, a large number of CSC inhibitors have proven that the elimination of CSCs is feasible. Despite these encouraging data, further actions are yet to be undertaken to optimize their application in combination with the conventional treatments under in vivo conditions and a clinical setting. Equally important, a broader understanding of the mechanisms regulating the immune-privilege status of CSCs would aid in devising strategies to disrupt their immunological attributes.

## Figures and Tables

**Table 1:** Small molecule CSC inhibitors in lung cancer.

Inhibitor	Mode of Action/Experimental Setting	Reference
HDAC11 (Histone deacetylase 11)	Reduced self-renewal property of NSCLC-derived CSCs; decreased SOX2 expression/in vitro	[94]
Salinomycin	Specifically targeted ALDH+ CSCs/in vitro	[95]
	Disrupted ALDH+ cells in A549-derived tumorspheres by decreasing Oct4, NANOG, and SOX2 expression levels in vitro	[96]
	Eliminated CSCs in metastatic LLC mouse model/in vivo	[97,98]
Aspirin (non-steroidal anti-inflammatory drug, NSAID)	Sensitized cisplatin-resistant NSCLC stem cells by targeting mTOR–AKT axis to repress cell migration/in vitro	[99]
	Reduced ALDH+ and SP cells; chemoresistance and sphere formation in lung cancer cells/in vitro; inhibited tumor growth, metastasis, and prolonged survival via a reduction in KDM6A/B expression mediating histone methylation that suppressed gene expression in a COX-independent manner/in vivo	[100]
Quercetin (Hsp 27 inhibitor)	Blunted activation of p38MAPK, MAPKAPK2, and Hsp27 after chemotoxic treatments; decreased survival of drug-resistant lung CSCs in combination with traditional chemotherapy/in vivo	[73]
Verrucarin J	Inhibited cell proliferation of CSCs; downregulates ALDH1, LGRs, Oct4, and CD133 via inhibition of the Wnt1/β-catenin and Notch1 pathways/in vitro	[101]
BBI608 (napabucasin)	Reduced ALDH+ CSC subpopulation by decreasing the mRNA levels of CSC-associated genes; had higher cytotoxic effects when combined with cisplatin; showed synergistic actions with paclitaxel/in vivo	[102]
	Suppressed the STAT pathway/in vitro; Phase 1b dose-escalation study in advanced solid tumors with napabusin plus weekly paclitaxel showed good toleration; Phase II study with napabusin and weekly paclitaxel in pretreated advanced NSCLC patients resulted in tumor regression, durable disease control, and prolonged progression overall survival	[103]
Nigericin	Inhibited cell viability of lung CSCs and resistance to anti-cancer agents; downregulated key proteins of the canonical Wnt signaling pathway/in vitro	[104]
MF-438 (SCDI inhibitor)	Restrained growth of cells with stem-like phenotype; reduced ALDH1 expression; impaired in vivo tumorigenicity/in vitro	[105]
	In combination with cisplatin, downregulated CSC markers; inhibited sphere formation and induced apoptosis/in vitro	[106]
Afatinib (EGFR inhibitor)	Ensued higher effectivity than cisplatin in enriched lung CSC subpopulations harboring EGFR mutations and in NSCLC primary cells expressing CD133/EpCAM/in vitro	[107]
AZD7762 (Chk1 inhibitor)	In combination with chemotherapy, significantly restrained NSCLC survival through modulation of premature cell cycle progression/in vitro; and reduced NSCLC CSCs in mouse xenografts/in vivo	[86]
GDC-0449 (Hedgehog inhibitor)	Reduced cell growth of HCC and H1339 lung cancer cells via suppression of SP cells/in vitro	[108]
DAPT (Notch1 inhibitor)	Inhibited cell growth of A549-derived CD44^+^/CD24^−^ subpopulation expressing high Notch1/in vitro	[109]
CPTH6 (histone acetyltransferase inhibitor)	Suppressed cell growth of lung cancer stem-like cells via induction of apoptosis/in vitro; inhibited tumor growth and reduced CSCs and tubulin acetylation in tumor xenografts/in vivo	[110]
IOX-101 (arylidene derivative)	Inhibited cell proliferation of A549 CSCs by increasing the sub-G0 cell cycle phase and rate of apoptosis; reduced MDR-1 and LRP expression; deactivated Akt and sub-G0 cell cycle/in vitro	[111]
Shisa3 (regulator of WNT and FGF signaling)	Controlled the growth of TKI-resistant PC9/ER xenografts and CSCs via interaction with FGFR_1_/3 to regulate the AKT/mTOR pathway/in vitro and in vivo	[112]
VS-5584 (dual PI3-mTOR inhibitor)	More potent than cisplatin and paclitaxel in eliminating CSCs in human cancer xenograft models; eliminated CSCs and delayed tumor regrowth in SCLC xenograft model after chemotherapy/in vivo	[113]

Abbreviations: NSCLC, non-small cell lung cancer; SCLC, small-cell lung cancer; CSC, cancer stem cell; EGFR, epidermal growth factor receptor; A549 and HCC, human lung adenocarcinoma cell lines; H1339, human lung carcinoma cell line; SOX2, SRY-Box transcription factor 2; ALDH, aldehyde dehydrogenase; ALDH1, aldehyde dehydrogenase 1 family, member A1; CSCs, cancer stem cells; Oct4, octamer binding transcription factor 4; NANOG, homeobox protein/transcription factor; MAPK, mitogen-activated protein kinase; MAPKPK2, mitogen-activated protein kinase-activated protein kinase 2; Hsp27, heat shock protein 27; LGRs, leucine-rich repeat-containing G protein-coupled receptors; CD133, cell differentiation 133, also known as prominin-1; STAT, signal transducers and activators of transcription; EGFR, epidermal growth factor receptor; Chk1, checkpoint kinase 1; EpCAM, epithelial cell adhesion molecule; SP, side population; SCDI, stearoyl-CoA desaturase; MDR-1, multidrug resistance protein 1; LRP, lung resistance-related protein; Akt, also known as serine/threonine protein kinase B (PKB); PI3, phosphoinositide 3-kinase; mTOR, mechanistic target of rapamycin; WNT, fusion name of Drosophila segment polarity gene *wingless* and the vertebrate homolog, integrated or int-1; FGF, fibroblast growth factor, CPTH6, (3-methylcyclopentylidene-[4-(4′-chlorophenyl) thiazol-2-yl] hydrazone); KDM6A/B, histone demethylase; COX, cyclooxygenase.

**Table 2:** Natural compound-based CSC inhibitors in lung cancer.

Inhibitor	Mode of Action/Experimental Condition	Reference
VF166 (isoflavone derivative of soy daidzein)	Inhibited cell proliferation, migration, and invasion of lung CSCs; regulated genes promoting cell invasion-related pathways, including Wnt-β-catenin, Hedgehog, STAT3, and SPARC/in vitro	[114]
Genistein (4′ 5, 7-trihydroxyisoflavone)	Inhibited cell viability and sphere-forming capacity and decreased protein expression of CD133, CD44, Bmi1, and Nanog in lung CSCs via regulation of MnSOD and FoxM1 expression levels/in vitro	[115]
Curcumin (diferuloylmethane) derived from *Curcuma longa*	Reduced self-renewal and sphere-forming abilities of lung CSCs via inhibition of DNA repair mechanisms/in vitro	[116]
	Suppressed colony and sphere-formation of lung CSCs through blockage of the JAK2/STAT pathway/in vitro	[117]
	Reduced CD133+ cells and other CSC markers; restrained cell proliferation and tumorsphere formation by inhibition of the Wnt/ß-catenin and Sonic Hedgehog pathways/in vitro	[118]
	Promoted sensitivity of CD166+/EpCAM+ lung CSC subpopulation to cisplatin through the p21 and cyclin D1-driven tumor cell inhibition/in vitro	[119]
Gigantol (extract from *Dendrobium draconis*)	Reduced sphere-forming ability and expression of CD133 and ALDH1A1; suppressed Oct4 and Nanog levels via inhibition of protein kinase B (Akt) activation/in vitro	[120]
Chrysotoxine (extract from *Dendrobium pulchenium*)	Restrained CSC phenotypes in H460 and H23 lung cancer cells via downregulation of Src/protein kinase B (Akt) signaling, that, in turn, depleted Sox-2 mediated-CSC phenotype/in vitro	[121]
Casticin (derivative of *Fructus viticis simplicifoliae*)	Suppressed self-renewal and cell proliferation of A549-derived lung CSCs; lowered protein levels of CD133, CD44, and ALDH1; decreased MMP-9 activity/in vitro	[122]
Renieramycin M (derivative of sponge *Xestospongia* species)	Reduced colony and sphere formation abilities of H460 CSCs; lowered expression of CD133, CD44, and ALDH1A1 in CSC-enriched H460 cells/in vitro	[123]
Silibinin (extract of *Silybum marianum*)	Decreased the percentage of stem cell-like ALDH^bright^ cells and self-renewal capacity of erlotinib-refractory NSCLC cells/in vitro	[124]
Vanillin (principal component of *Vanilla planifolia* seeds)	Restrained spheroid and colony formation; controlled CD133, ALDH1A1, Oct4, and Nanog at low levels in H460 lung cancer cells via induction of Akt-proteasomal degradation and reduction of downstream CSC transcription factors/in vitro	[125]
BRM270 (extract from seven herbal plants)	Regulated A549 CSCs’ self-renewal property and their ability to initiate tumor through regulation of the miRNA-128; decreased cell proliferation and mediated apoptosis in drug-refractory A549 through regulation of VEGF/PI3K/AKT signaling via miR-128/in vitro and in vivo	[126]
Chetomin (extract of *Chaetonium globosum*)	Decreased sphere-forming capacity and stem cell-like phenotypes of NSCLC CSCs by blocking the heat shock protein 90/hypoxia-inducible factor-alpha (Hsp90/HIF1α) signaling activity/in vitro	[127]

Abbreviations: NSCLC, non-small cell lung cancer; CSC, cancer stem cell; A549 and H23, human lung adenocarcinoma cell lines; H460, human lung carcinoma cell line; SOX2, SRY-Box transcription factor 2; ALDH1A1, aldehyde dehydrogenase 1 family, member A1; CSCs, cancer stem cells; Oct4, octamer binding transcription factor 4; NANOG, homeobox protein/transcription factor; CD133, cell differentiation 133, also known as prominin-1; JAK2, Janus kinase 2; STAT, signal transducers and activators of transcription; STAT3, STAT protein 3, EpCAM, epithelial cell adhesion molecule; Bmi1, polycomb complex protein; SPARC, secreted protein acidic and rich in cysteine; MMP-9, matrix metallopeptidase 9; NF-κB, nuclear factor “kappa-light-chain-enhancer” of activated B-cells; VEGF, vascular endothelial growth factor; PI3K, phosphoinositid-3-kinase; AKT, also known as protein kinase B or PKB; miR-128, microRNA-128; Casticin,5,3′-dihydroxy-3,6,7,4′-tetramethoxyflavone; MnSOD, manganese superoxide dismutase; FoxM1, Forkhead Box M1.
